# A case report of intravenous posaconazole in hepatic and renal impairment patient with invasive *Aspergillus terreus* infection: safety and role of therapeutic drug monitoring

**DOI:** 10.1186/s40360-017-0115-z

**Published:** 2017-01-31

**Authors:** Pitchaya Dilokpattanamongkol, Panadda Panusitthikorn, Rasda Boonprasert, Methee Chayakulkeeree, Porpon Rotjanapan

**Affiliations:** 1 Department of Pharmacy, Faculty of Pharmacy, Mahidol University, 447 Sri-Ayutthaya Road, Ratchathewi Bangkok, 10400 Thailand; 2 Department of Pharmacy, Faculty of Medicine, Siriraj Hospital, Mahidol University, 2 Wanglang Road Bangkoknoi, Bangkok, 10700 Thailand; 3 Clinical Toxicology Laboratory, Siriraj Poison Control Center, Faculty of Medicine, Siriraj Hospital, Mahidol University, 2 Wanglang Road Bangkoknoi, Bangkok, 10700 Thailand; 4 Division of Infectious Diseases and Tropical Medicine, Department of Medicine, Faculty of Medicine, Siriraj Hospital, Mahidol University, 2 Wanglang Road Bangkoknoi, Bangkok, 10700 Thailand; 5 Division of Infectious Diseases, Department of Medicine, Ramathibodi Hospital, Mahidol University, 270 Rama VI Road, Ratchathewi Bangkok, 10400 Thailand

**Keywords:** Case report, Invasive aspergillosis, Posaconazole, Safety, Therapeutic drug monitoring

## Abstract

**Background:**

Invasive aspergillosis (IA) is a fatal infectious complication among immunocompromised patients. *Aspergillus terreus*, the fourth common species can be difficult to treat due to a unique resistance pattern. To date, there has been no report on safety and dose adjustment when intravenous posaconazole is selected in hepatic and renal impairment patient. We present a rare case of intravenous posaconazole use in a hepatic and renal impairment patient with invasive *A. terreus* pulmonary infection. To our knowledge, this is the first report of intravenous posaconazole use in IA due to *A. terreus* with hepatic and renal impairment focusing on drug safety and role of therapeutic drug monitoring (TDM).

**Case presentation:**

A 37-year-old previously healthy man with diagnosis of dengue hemorrhagic fever and shock complicated with hepatic and renal impairment proposed to have proven invasive *A. terreus* pulmonary infection is described. Due to lack of good clinical response and concern of potential adverse effects whilst on intravenous voriconazole, intravenous posaconazole 300 mg every 48 h was chosen with confirmed therapeutic plasma concentrations. Despite the death of the patient and IA deemed uncontrollable, there were no significant side effects attributable to intravenous posaconazole use demonstrated over a period of 34 days.

**Conclusions:**

Intravenous posaconazole use with TDM implementation maybe a safe alternative option to standard therapy. Therapeutic plasma posaconazole level may be reached at lower dosing regimen in renal and hepatic impairment patient. However, explanations of clinical failure on this patient with immunodeficiency state were multifactorial.

**Electronic supplementary material:**

The online version of this article (doi:10.1186/s40360-017-0115-z) contains supplementary material, which is available to authorized users.

## Background

Invasive fungal infections (IFIs) due to *Aspergillus* species have become a major cause of morbidity and mortality, especially in immunocompromised patients [1]. *A. terreus* has been reported to be the fourth common species isolated according to Antifungal Surveillance Program in 2011 preceding by *A. fumigatus*, *A. flavus*, and *A. niger* [2].

IA has been estimated to occur in 5%-40% of hematologic patients, especially in acute myeloid leukemia and allogeneic hematopoietic stem cell transplant with mortality rate attributable to IA at 25%–50% with standard antifungal regimen available [3, 4]. Treatment option for IA secondary to *A. terreus* may be limited mainly to triazole antifungal group given high level of resistance to amphotericin B either from in vivo or in vitro data [5]. Hence, in some instance when hepatic function is impaired, treatment for this particular IA can be more difficult [6].

Current guidelines by the Infectious Diseases Society of America in the year of 2016 recommends voriconazole as first-line therapy [7]. Voriconazole has fungicidal effect to common strains of *Aspergillus* spp. but has been reported to play a fungistatic effect against *A. terreus* particularly with wide range of minimum inhibitory concentrations (MICs) from 0.125 mg/L to 32 mg/L [8]. Key indicator to good treatment outcome is TDM which is true in all triazole drugs [9]. Therefore, in order to obtain adequate level in critically ill patients, parenteral route is preferred so that gastrointestinal absorption issue is out of concern [10]. However, the parenteral formulations of both voriconazole and posaconazole contain sulphobutylether-b-cyclodextrin (SBECD) as excipients for solubilization of drug with greater proportion in voriconazole that have either been reportedly associated with hepatotoxicity or nephrotoxicity [11, 12]. And more importantly, both renal and hepatic dysfunctions are not uncommon in critically ill patients at baseline [10] in which intravenous use of voriconazole should be carefully considered. However, intravenous voriconazole use is allowed in real clinical practice to some period of time in renal insufficiency patients without serious adverse side effects [13] but essentially deems insufficient for cure of IA.

Posaconazole is recognized as an effective substitute in case of treatment failure of voriconazole for IA [7]. Hence, intravenous posaconazole may be a good option for IA treatment when MIC is noted to be less than 0.25 mg/L and plasma concentration above 1.0 mg/L in the setting that renal and/or liver impairment would be a limitation to voriconazole use [14, 15]. However, data on drug safety and role of TDM on parenteral posaconazole use is still lacking in patients with renal and hepatic impairment. Here, we present a rare case of intravenous posaconazole use in critically ill patient with invasive *A. terreus* pulmonary infection and renal and hepatic impairment who had been admitted to Ramathibodi Hospital, Mahidol University, Bangkok, Thailand between November, 2015 and January, 2016 for new insight of this antifungal.

## Case presentation

### Hospital course, bacterial infections, and treatment complications

A previously healthy 37-year-old male was referred to our hospital with a diagnosis of dengue hemorrhagic fever with shock. His clinical course began 6 days prior to transfer when he presented to an outside hospital with fever. He was found to have left sided hemothorax on arrival causing respiratory distress in which intercostal drainage was promptly placed. However, his condition remained unstable following the procedure requiring intravenous norepinephrine for hypotension. His clinical course was shortly complicated by acute renal failure necessitating renal replacement therapy via right internal jugular catheter on hospital day 2. Despite supportive treatment, lactic acidosis was newly demonstrated together with the need for higher dose of vasopressive drug, various antibacterial regimens were prescribed throughout hospitalization and meropenem and vancomycin were selected as initial combination.

On hospital day 3, due to progressive respiratory distress and failure on lung recruitment maneuver, extracorporeal membrane oxygenation (ECMO) was initiated via right femoral catheter. He was also noted to have pancytopenia. Bone marrow study findings were compatible with hemophagocytosis syndrome secondary to dengue infection. Intravenous dexamethasone (10 mg/m^2^) and intravenous immunoglobulin were started and plasmapheresis replaced immunoglobulin therapy on the following day. Hematologist was able to reverse pancytopenic event 7 days later but profound lymphopenia persisted throughout his hospital course with the highest absolute CD4 count of 90 cells/mm^3^.

On hospital day 11, ECMO was discontinued and right femoral catheter was removed after stabilization of overall condition. However on hospital day 13, he underwent left below knee amputation for better control of limb ischemia presumably secondary to ECMO complication that had been documented since hospital day 8. His clinical course was also complicated by 1.) Cytomegalovirus reactivation during dexamethasone therapy and required brief duration of ganciclovir treatment given no specific organ involvement identified and 2.) *Chromobacterium viloaceum* pneumonia and secondary bacteremia on hospital day 18. Bacterial pneumonia was very difficult to control that he required a prolonged course of antibiotics consisting of piperacillin/tazobactam and ciprofloxacin to the point that left pneumonectomy was indicated on hospital day 46. After the surgery, he was prescribed several short courses of antibiotic treatment mainly in order to prevent gut microbial translocation following numerous events of gastrointestinal bleeding. Hemorrhagic complication was later discovered to be secondary to large stress associated rectal ulcer. He died on hospital day 71 due to massive esophageal bleeding that autopsy findings failed to identify infectious etiology at the bleeding site.

### Proven invasive pulmonary aspergillosis

Regarding fungal infection risk particularly IA following severe dengue infection, micafungin was begun on hospital day 3 after serum galactomannan (GM) was obtained which later reported to be 2.3 by enzyme-linked immunosorbent assay (ELISA). Liposomal amphotericin B replaced micafungin for new diagnosis of probable IA that chest radiograph now displayed faint opacities in right middle lung field. In addition, anidulafungin was added in response to rapid progression of pulmonary lesions on chest radiograph. After 6 days of combination therapy, serum GM level declined to 0.55 as the lowest value but no change on chest radiograph findings and respiratory status.

On hospital day 18, micafungin replaced anidulafungin in light of possible cardiac toxicity. Bronchoscopy was done to determine the potential pathogenic fungi but biopsy was avoided given very compromised respiratory status. Antifungal regimen had been switched to and fro between liposomal amphotericin B and amphotericin B deoxycholate due to a fear of drug induced cholestatic jaundice from hospital day 21 until day 31 when polyene antifungals were halted completely after a recognition of *A. terreus* growth on every respiratory specimen. Intravenous and nebulized voriconazole and oral flucytosine via nasogastric tube were now chosen. Despite of therapeutic voriconazole levels in the range of 3.29–4.89 mg/L, pulmonary lesions were still growing in size with increment of serum GM to 9.73 and worsening respiratory parameters over a period of 10 days. A decision to switch treatment to intravenous posaconazole was made in view of potential SBECD accumulation. Plasma levels of posaconazole were measured to assure drug exposure adequacy. MICs of current *A. terreus* infection to voriconazole, posaconazole, caspofungin, anidulafungin, micafungin were 0.75, 0.125, 1, 0.004, and 0.064 mg/L, respectively.

Determination of posaconazole concentrations in plasma was performed using validated Ultra Performance Liquid Chromatography-Photo Diode Array (UPLC/PDA), according to the US Food and Drug Administration guidance for bio-analytical method validation [16]. Blood samplings for plasma posaconazole levels were performed dividing into two phases. The first phase took place when posaconazole 300 mg was given every 24 h, spotted plasma levels were obtained to determine the appropriate timing of the following doses in which resulted in a new dosing regimen of every 48-h interval. In the second phase happened when posaconazole 300 mg was given every 48 h, blood samples were collected for pharmacokinetic analysis at 0 h (predose), immediately at the end of infusion, approximately 15 min after the end of infusion, and approximately 4, 8, 12, 24 and 48 h after the start of infusion. After 34-day course of posaconazole with confirmation of therapeutic plasma posaconazole levels almost the entire period of time during posaconazole treatment (0.956–7.099 mg/L), his infection still did not subside. Serum GM prior to his death was 8.97. Autopsy result confirmed diffuse IA in his right lung without evidence of dissemination to other organs as well as no bacterial pneumonia documented. Additional immunological tests also declared impairment of natural killer T-cell function. Plasma posaconazole levels and serum GM are shown in Additonal file 1: Figure S1. An additional figure file shows summary of his clinical course [see Additional file 1].

Liver function tests and QTc interval on electrocardiography data prior and during posaconazole treatment are displayed in Table 1. The findings demonstrated a decline in aspartate transaminase (AST) and alanine transferase (ALT) levels significantly after posaconazole use (*p* =0.005 and 0.028, respectively). On the other hand, there was an increase in alkaline phosphatase (ALP) and gamma-glutamyl transpeptidase (GGT) levels after posaconazole use with statistical significance (*p* = <0.001 and 0.039, respectively). There were no crucial changes on total bilirubin (TB), direct bilirubin (DB) and QTc interval. Statistical analyses on relevant parameters to compare the differences prior and during posaconazole therapy were performed using SPSS version 21.0 for Windows (IBM Corp., Armonk, New York). The distribution of continuous variables was determined by Kolmogorov-Smirnov test. All continuous variables were assessed by the Student's t-test or Wilcoxon Rank Sum test as appropriate. For normally distributed variables, data were described by mean ± standard deviation (SD). For skewed variables were described as median (range). A *p* value of less than 0.05 was considered significant for all statistic values.

**Table 1:** Liver function tests and QTc interval (pre and during posaconazole treatment)

	Pre-posaconazole treatment	During posaconazole treatment	*p*-value
Aspartate transaminase (median, range)	210, 64-982 units/L	109, 48-375 units/L	0.005
Alanine transferase (median, range)	223, 69-1060 units/L	121, 78-409 units/L	0.028
Alkaline phosphatase (median, range)	143, 60-323 units/L	286, 180-478 units/L	<0.001
Gamma-glutamyl transpeptidase (median, range)	104, 33-341 units/L	146, 84-421 units/L	0.039
Total bilirubin (mean+/-SD)	22.38+/-10.45 mg/dL	19.57+/-3.42 mg/dL	0.124
Direct bilirubin (median, range)	16.20, 1.50-26.00 mg/dL	13.30, 9.10-16.30 mg/dL	0.217
QTc interval(median, range)	0.435, 0.350-0.490 s	0.450, 0.400-0.490 s	0.060

## Discussion

Invasive *A. terreus* infection is considered a fatal infection given its poor prognosis [17]. One of the explanations to this fact is unique resistance pattern of the fungus that distinguishes *A. terreus* from other species making polyene antifungal agent not an option [18]. Voriconazole remains the mainstay of treatment of most IA [7] except in a rare instance that precludes individuals from voriconazole use such as pre-existing liver condition or renal impairment that can be a risk of SBECD accumulation particularly intravenous formulation [11].

Posaconazole is conceivably an alternative treatment for IA and also recommended in salvage therapy [7]. Hepatic failure has been rarely reported with posaconazole use [19]. On the other hand, this complication can be found from 2.7% up to 12.4% with voriconazole prescription [20] . SBECD, as a potentially nephrotoxic agent comprising in intravenous triazoles has been revealed recently in numerous clinical studies either in human or animal models not to be a significant threat when recommended dosing is delivered but no data on long-term use thus far [11, 13, 21]. Hence, a concern of antifungal composition causing adverse events may not be a major consequence and posaconazole may be a good option among hepatic and renal impaired individuals given the least SBECD component comparing to other triazoles [12].

Similarly to voriconazole and other agents, TDM is suggested in routine clinical practice [9]. Walsh TJ, et al recommended to maintain plasma posaconazole level above 1 mg/L at all time for treatment success [22] but to date, there have not been any data on plasma posaconazole level in correlation with drug safety. Given an emergence of less common *Aspergillus* spp. including *A. terreus*, specific recommendation to support posaconazole use in order to avoid adverse drug events in hepatic and renal impairment is still lacking.

To our knowledge, this is the first case report that focuses on an association of plasma posaconazole levels and safety in invasive *A. terreus* patient that had both hepatic and renal impairment. We were able to maintain therapeutic plasma posaconazole levels at all time. However, clinical failure was observed in this patient describing as deterioration of respiratory status, worsening of pulmonary radiographs, and persistent isolation of the fungus both pre-and postmortem. The plausible explanations include unrevealing myth about posaconazole in some aspect important to clinical practice especially in similar circumstance and factors unrelated to antifungal therapy. Fundamentally, key indicators to microbiological and clinical success besides optimal supportive measures constitute of at least two elements [23]. Appropriate antimicrobial treatment is the cornerstone. All steps of antimicrobial administration should be taken into account starting from agent selection, appropriate dosing regimen, route of administration, clinical efficacy evaluation, adverse effect awareness, or even therapeutic drug monitoring [24]. The other important component is intact host immune response [25]. Diminished number and/or function of white blood cells including CD4 count has long been realized as a major risk of fungal infection as well as difficult to control of disease [26]. Our patient lost his ability to maintain good immune response both innate and adaptive immune response based on immunological investigations. Therefore, one key component to fungal clearance was missing in which clinical failure can be easily predicted.

We initiated recommended dose of intravenous posaconazole 300 mg daily in the beginning and monitored plasma levels intermittently to assure optimal level. We discovered that at some points, plasma levels rose above 5-7 mg/L persistently which were 5–7 times higher than desired level [9]. Regarding safety concerns, our patient was documented to have liver impairment prior to posaconazole initiation but no significant further deterioration of hepatic parameters but in fact a decline in AST and ALT levels although ALP and GGT were trending up prior to his death. On electrocardiography monitoring throughout hospitalization, QTc prolongation was not an issue as well (0.400–0.490 s) indicating intravenous posaconazole was safe in this aspect to some extent. Nevertheless, the dose of posazonazole was adjusted on several occasions from every 24–120 h. The final dosing regimen was 300 mg every 48 h that we were able to maintain plasma trough levels in the range of 0.946–2.838 mg/L at all time. This might be a hint to future practice that hepatic impaired patient may not require standard once daily dosing but rather less frequent administration given plasma levels were elevated higher than expected and without other logical explanation. Slower hepatic metabolism, especially in critically ill patients, than in normal hepatic function is a possibility [27].

Based on these findings, we may conclude that intravenous posaconazole use may be safe in pre-existing hepatic and renal impairment individuals undergoing renal replacement therapy. The manufacturer’s suggested dosing regimen of posaconazole in renal and liver impairment patients is presented in Table 2.

**Table 2:** Recommended dose for posaconazole in renal and liver impairment patients [12]

Population	Posaconazole dose adjustment
Renal impairment	
Creatinine clearance 20-80 ml/min	No adjustment necessary
Creatinine clearance < 20 ml/min	No adjustment necessary, monitor for breakthrough fungal infections due to variability in exposure
Hepatic impairment	
Child-Pugh A, B, C	No adjustment necessary

However, these are based on a single case observation on hepatic and renal impairment taking intravenous posaconazole treatment. This maybe too premature to conclude that posaconazole is safe in every hepatic and renal impaired patients with/without dose adjustment but potentially such patients can reach therapeutic level with lower dosing regimen. Hence, a study at a larger scale is encouraged to verify these observations.

## Conclusions

Intravenous posaconazole use at conventional dose in critically ill patients with reduced liver and renal functions may cause unexpectedly elevated posaconazole plasma level but potential adverse events were not observed in correlation with presumptive “supra-and therapeutic values” in this case report. Reduction of maintenance dosage with guidance of TDM may be necessary in order to avoid unaware adverse effects. However, posaconazole therapy was not a success in this case study and the explanation is likely multifactorial.

## Additional file

Additional file 1: Figure S1. Course of antifungal therapy, interventions, serum GM and plasma posaconazole levels. (XLSX 369 kb)

## Abbreviations

ALP: Alkaline phosphatase
ALT: Alanine transferase
AST: Aspartate transaminase
cells/mm^3^: cells per milliliter
ClCr: Creatinine clearance
DB: Direct bilirubin
ECMO: Extracorporeal membrane oxygenation
g/dL: gram per deciliter
GGT: Gamma-glutamyl transpeptidase
GM: Galactomannan
IA: Invasive aspergillosis
IFI: Invasive fungal infection
mg/dL: milligram per deciliter
mg/L: milligram per liter
mg/m^2^: milligram per square meterNK: natural killer
MIC: Minimum inhibitory concentration
SBECD: Sulphobutylether-b-cyclodextrin
TB: Total bilirubin
TDM: Therapeutic drug monitoring
